# SNP rs4937333 in the miRNA-5003-Binding Site of the *ETS1* 3′-UTR Decreases *ETS1* Expression

**DOI:** 10.3389/fgene.2019.00581

**Published:** 2019-06-19

**Authors:** Ruixian Zhang, Bangpin Pan, Yi Li, Xiaolan Li

**Affiliations:** ^1^Department of Dermatology, The Second Affiliated Hospital of Kunming Medical University, Kunming, China; ^2^Yunnan Center for Disease Control and Prevention, Kunming, China

**Keywords:** systemic lupus erythematosus, *ETS1*, single-nucleotide polymorphism, B cell, miRNA, differentiation

## Abstract

Mutations in and reduced expression of the *ETS1* gene may be associated with systemic lupus erythematosus (SLE). Here, we report a replication study to investigate associations of eight *ETS1* single-nucleotide polymorphisms in the 3′-untranslated region (3′-UTR) with SLE and their regulation of *ETS1* expression in a study population. We found that the rs4937333 T allele was associated with a significantly increased risk of SLE (odds ratio: 1.800, 95% confidence interval: 1.02–3.157, *P* = 0.040) and with dramatically reduced levels of *ETS1* in B cells from SLE subjects. Functionally, the rs4937333 T allele alters the binding affinity between miR-5003 and its *ETS1* 3′-UTR target, thus enhancing suppression of *ETS1* expression. Furthermore, immunoglobulin M-secreting plasmacytes were significantly reduced among B cells with the rs4937333 C allele versus the T allele according to FACS and ELISA. Additionally, miR-5003 expression was higher in B cells than in T cells from SLE patients, and a negative correlation between miR-5003 and *ETS1* was found, especially in B cells with the T allele. These findings suggest that the rs4937333 T allele is a risk factor for susceptibility to SLE in the studied population. The rs4937333 T allele may enhance the binding of miR-5003 to *ETS1*, which probably promotes the involvement of *ETS1* in the differentiation of B cells into plasmacytes.

## Introduction

Systemic lupus erythematosus (SLE) is a complex, fatal, and heterogeneous chronic autoimmune disease (Carter et al., 2016). SLE pathogenesis includes abnormal T and B cells, aberrant autoantibody production, and dysregulated cytokine production (Anolik, 2007; Jacob and Stohl, 2011). Studies on both humans and mouse models of the disease have shown that genetic factors strongly contribute to the pathogenesis of SLE (Crampton et al., 2014). Genome-wide association studies (GWASs) have identified some SLE susceptibility genes, highlighting potential disease mechanisms (Mohan and Putterman, 2015).

One SLE-associated gene encodes the transcription factor *ETS1*, which is a member of the ETS family of transcription factors and plays a role in lymphocyte development and function, apoptosis, and inflammation (Pan et al., 2011). Over-activated B cells differentiate into plasma cells have an important role in the pathogenesis of SLE by producing anti-nuclear autoantibodies (including specific antibodies that bind to double-stranded DNA) and presenting autoantigens to T cells (Nashi et al., 2010; Malkiel et al., 2018). *ETS1* is a negative regulator of B cell differentiation, and *ETS1* deficiency is strongly correlated with an increase in immunoglobulin M (IgM)- and IgG-secreting plasma cells (Wang et al., 2005). Previous studies have found that *ETS1* mRNA levels are lower in peripheral blood mononuclear cells (PBMCs) from SLE patients than in PBMCs from healthy controls (Yang et al., 2010). However, the *ETS1* expression patterns in B cells and CD4+ T cells remain unclear, and the mechanism of *ETS1* dysregulation is unknown.

Several studies have revealed the effects of genetic variants that decrease *ETS1* expression in autoimmune diseases (Deng and Tsao, 2010; Yang et al., 2010; Shan et al., 2014). Evidence indicates that several single-nucleotide polymorphisms (SNPs) in the 3′-untranslated region (UTR) of *ETS1* were associated with SLE (Han et al., 2009; Yang et al., 2010; Armstrong et al., 2014; Shan et al., 2014; Lessard et al., 2016). MicroRNAs (miRNAs) are non-protein-coding RNA molecules that regulate the translation and degradation of messenger RNA (mRNA) through sequence complementarity with 3′-UTR regions; such regulation has been implicated in a wide range of biological processes, including cell proliferation, differentiation, and death (Schickel et al., 2008; Catalanotto et al., 2016). SNPs in miRNA target sites in the 3′-UTRs of mRNA molecules represent a specific class of functional polymorphisms that may lead to dysregulation of posttranscriptional gene expression by disrupting regulatory miRNA binding. An increasing number of 3′-UTR SNPs in miRNA-binding sites has been found to be associated with SLE, including rs1057233 in the miR-569-binding site of SPI1 (Hikami et al., 2011) and rs3853839 in the miR-3148-binding site of toll-like receptor 7 (*TLR7*) (Deng et al., 2013). However, whether SNPs in the 3′-UTR of *ETS1* are associated with SLE in the Chinese population remains unclear.

We hypothesized that SNPs in the 3′-UTR of *ETS1* may change miRNA binding to *ETS1*, contributing to the disordered expression of the gene. In the present study, we found that rs4937333 was associated with an increased risk of SLE in the Han population in the Kunming area. The T variant of rs4937333 affected the binding affinity of miR-5003 to the 3′-UTR of the *ETS1* gene, thus disrupting posttranscriptional regulation of the gene and resulting in enhanced differentiation of B cells into plasma cells.

## Materials and Methods

### Subjects

We recruited 108 unrelated subjects for this case–control study from January to October 2018, including 66 patients with SLE (62 females, 93.9%) from the Second Affiliated Hospital of Kunming Medical University. All patients, who had a mean age of 36 ± 7.3 years, met the modified criteria for SLE (Petri et al., 2012). We also recruited 42 unrelated, randomly sampled healthy controls (39 females, 92.9%) from a health check-up center at the hospital during the same period; the mean age of these subjects was 38 ± 8.2 years. The inclusion criteria for SLE were as follows: A Systemic Lupus Erythematosus Disease Activity Index (SLEDAI-2K) < 3, reflecting the stationary phase, and the use of only a low-maintenance dosage of prednisone within the past 3 months (Gladman et al., 2002). The protocol for this research was approved by the Ethics Committee of the Second Affiliated Hospital of Kunming Medical University, and all subjects in this study provided informed consent.

### Isolation and Culture of PBMCs, B Cells, and CD4+ T Cells

Peripheral blood mononuclear cells were purified from heparinized venous blood by density gradient centrifugation with Lymphoprep (Axis-Shield, Oslo, Norway). Density gradient centrifugation was performed for 20 min at 400 × *g* and 18–20°C within 2 h after blood specimens were obtained. CD4+ cells were enriched by positive selection with CD4 magnetic microbeads (Miltenyi Biotec, Bergisch Gladbach, Germany) according to the manufacturer’s protocol. To obtain highly purified CD4 cells, the PBMCs were incubated with the microbeads (20 μL/10^7^ cells) at 4°C for 15 min and then passed through LS columns using a QuadroMACS separator (Miltenyi Biotec). During this process, degassed buffer containing phosphate buffer saline (PBS, pH 7.2), bovine serum albumin (BSA, 0.5%), ethylenediaminetetraacetic acid (EDTA 2 mM) was used to wash the columns. The same procedure was used to isolate CD19+ lymphocytes from the remaining cells according to the manufacturer’s instructions.

### SNP Selection and Genotyping

We selected eight SNPs (rs1128355, rs11554584, rs12288765, rs35034701, rs58920409, rs57498864, rs1128334, and rs4937333) in the 3′-UTR of *ETS1* based on published studies (Han et al., 2009; Yang et al., 2010; Guo et al., 2013) and prediction results from miRdSNP (Bruno et al., 2012) and PolymiRTS Database 3.0 (Bhattacharya et al., 2013). Genomic DNA was extracted from blood leucocytes using a whole-blood genomic DNA extraction kit (Tiangen, China) following the manufacturer’s instructions. The gene polymorphisms were analyzed using PCR sequencing.

### Quantitative PCR for *ETS1*

Total RNA was extracted from PBMCs, B cells, and T cells from 66 SLE patients and 42 healthy controls by the TRIzol reagent method (Invitrogen, United States) and then reverse transcribed into cDNA with a reverse transcription kit (Takara, Japan). Allele-specific expression of *ETS1* was analyzed only in healthy controls. Real-time quantitative PCR (RT-qPCR) was performed to amplify cDNA with a Roche 480 RT-PCR system with SYBR Green Master Mix (Kapa Biosystems, United States). Relative expression was analyzed by the comparative threshold cycle (Ct) method and normalized to the expression of human glyceraldehyde-3-phosphate dehydrogenase (GAPDH). Expression values were calculated by the 2^–Δ^ method and log 10 transformed. The primer sequences used for genotyping of the SNPs and qPCR of miRNA or *ETS1* are shown in Supplementary Table S1. Melting curve analysis was used to confirm specificity, and three replicate wells were used for each subject (Schmittgen and Livak, 2008). Extracted total RNA was also subjected to RT-PCR to detect miRNA levels in subjects with and without the variant allele using an Ambion RT-PCR kit and a QuantStudio 12K Flex RT-PCR System.

### Vector Construction

A 337-bp fragment containing the rs4937333 locus on the 3′-UTR of *ETS1* was amplified by PCR from two homozygous patients (carrying TT or CC genotype) using the following primers: sense (5′-GCCTCTTGCTTGGGTCTGAT-3′) and antisense (5′-AAGCCACCCCTCCTCCTTAT-3′). The PCR product was subcloned into the SacI and XhoI site of firefly luciferase in the pGL3 vector (Promega, Biloxi, MS, United States).

To investigate the effect of a T mutation at the rs4937333 site in the human *ETS1* gene *in vitro*, the human *ETS1* ORF was cloned into the pEZ-LV201 vector (GeneCopoeia, Inc., Rockville, MD, United States), and the 337-bp fragment of the *ETS1* 3′-UTR containing either a T or C allele at the rs4937333 locus was inserted downstream of the human *ETS1* gene. All constructs were confirmed by sequencing.

### Lentiviral Production and Transduction

For lentivirus production, the Lenti-Pac 293Ta packaging cell line was transfected with various lentivirus expression plasmids using the Lenti-Pac HIV Expression Packaging Kit (GeneCopoeia, Inc., Rockville, MD, United States). Lentiviral supernatants were used to infect 3 × 10^6^ B cells, which were stimulated with 5 μg/ml CpG ODN (Class B, invivoGen, San Diego, CA, United States). Two days after infection, the GFP-positive population was sorted out using the FACsAria Cell Sorter (BD Biosciences Immunocytometry Systems). The resulting data were analyzed on a BD LSRFortessa^TM^ cell analyzer using FlowJo software 6.0 (TreeStar Inc., Ashland, OR, United States).

After 48 h of incubation in CpG ODN, the supernatants were harvested, and ELISA was carried out using a commercial kit (NeoBioscience, China). The proliferation of sorted GFP+ virally infected cells was measured by CFSE assays as previously described (Aab et al., 2017).

### Thermodynamic Model for Predicting miRNA–Target Interactions

We used MirSNP, the PolymiRTS Database 3.0, and MicroSNiPer to predict the SNP rs4937333 located in the seed region of the miR-5003 target sites of *ETS1*. To investigate the binding affinity of miR-5003 to the target sites with rs4937333 variants, we used a 60-bp region (containing the seed site) to calculate the energies of the secondary structures using a parameter-free thermodynamic model. Briefly, RNAFold (Vienna RNA Package) was used to compute *E*_target_, which is the secondary structure of the target with the lowest free energy based on its sequence. RNAcoFold was used to calculate *E*_intermediate_ and *E*_complex_. *E*_intermediate_ is the local energy of the transition state when we force the binding site to be unpaired, and *E*_complex_ is the local energy of the miRNA and target mRNA dimer (Zhang et al., 2011).

### miRNA Mimic Transfection

An miR-5003 mimic and negative control mimic were ordered from RiboBio Company (Guangzhou, China). The miRNA mimics were transfected into 293T cells and B cells for luciferase reporter assays and Western blot assays after 48 h of transduction as described below.

### Luciferase Reporter Gene Assay

To analyze luciferase activity, 293T or B cells were seeded in 24-well culture plates 24 h before transfection. The miRNA-5003 mimic or control mimic was cotransfected with reporter vectors (pGL3 control empty vector or the rs4937333 T or C allele constructs) using Lipofectamine 2000 (Invitrogen, United States) following the manufacturer’s protocol. The vector pRL-SV40 (Promega, Biloxi, MS, United States) was used as an internal control. Firefly and Renilla luciferase activity levels were measured at 24 h after transfection using a Dual-Glo Luciferase Assay System (Promega, Biloxi, MS, United States). Relative reporter activity was obtained by normalization to Renilla luciferase activity (the ratio of firefly luciferase activity to Renilla luciferase activity). The experiments were independently performed in triplicate.

### Western Blot Analysis

Whole-cell lysate was prepared using RIPA lysis buffer (Beyotime Biotechnology, China). Protein concentrations were determined by BCA quantification using a BCA protein assay kit (Tiangen, China). The samples were subjected to 10% SDS–PAGE and transferred to PVDF membranes (Millipore, United States). After blocking at room temperature for 2 h, the membranes were incubated at 4°C overnight with rabbit polyclonal anti-*ETS1* (1:1000 dilution, clone C-20, Santa Cruz Biotechnology, Santa Cruz, CA, United States) and mouse monoclonal anti-β-actin (1:2000 dilution, clone C-20, Santa Cruz Biotechnology, Santa Cruz, CA, United States). Secondary antibodies labeled with horseradish peroxidase (1:5000 dilutions, Santa Cruz Biotechnology, Santa Cruz, CA, United States) and an ECL kit (Pierce, United States) were used to detect the protein signals. The Western blot bands were quantified by scanning densitometry using Quantity One software (Bio-Rad). All experiments were performed at least three times.

### Flow Cytometry

Flow cytometry was performed to investigate the effects of SNPs on B cell differentiation into plasma cells. Briefly, lentivirus-transduced cells were stained with phycoerythrin-conjugated anti-mouse Syndecan-1 (CD138) (BD Biosciences Pharmingen). The samples were analyzed on a BD Biosciences Immunocytometry Systems FACSCalibur flow cytometer, and the resulting data were evaluated using FlowJo software 6.0 (TreeStar Inc., Ashland, OR, United States).

### Elisa

Equivalent numbers of sorted GFP+ cells from the lentivirally transduced populations were resuspended in medium containing 5 μg/ml CpG ODN. After 48 h, the supernatants were harvested, and ELISA was carried out using a commercial kit (NeoBioscience, China).

### Statistical Analysis

The SNPs were tested for adherence to Hardy–Weinberg equilibrium (HWE) by the Chi-squared test. The allele frequencies of the two SNPs in patients and controls were compared using SPSS19.0 with Fisher’s exact test. Differences in allelic expression were analyzed by *t*-tests and analysis of variance (ANOVA) with GraphPad Prism v6.0 (GraphPad Software, United States). The relationship between *ETS1* transcript levels and miR-5003 was evaluated using linear regression. *P* < 0.05 was considered statistically significant.

## Results

### Association Study

**Figure 1:**
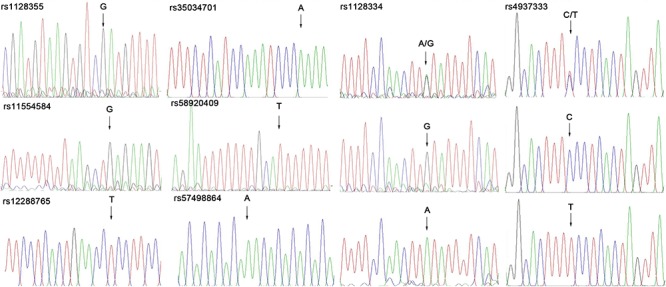
Use of sequencing technology to genotype the SNP in *ETS1*.

We investigated the genotypes and allele distributions of two SNPs in *ETS1* in 42 controls and 66 patients with SLE in the Kunming area (Figure 1). Neither SNP had an allele frequency that deviated significantly from HWE at the 0.05 significance level. The frequency of the rs4937333 T allele was significantly higher in SLE patients [*P* = 0.040, odds ratio (OR): 1.800, 95% confidence interval (CI): 1.026–3.157] than in healthy controls. The other SNPs were not associated with SLE (Table 1).

**Table 1:** Genotype and allele association analysis of SNPs in the *ETS1* 3′-UTR in the Kunming population.

SNPs	Genotype/allele	SLE, *n* (%)	control, *n* (%)	Odds ratio	95% CI	*P*-value
rs1128334	GG	39 (59.1)	30 (71.4)			
	GA	18 (27.3)	9 (21.42)	1.538	0.606–3.903	0.364
	AA	9 (13.6)	3 (7.14)	2.308	0.574–9.271	0.239
	G	96 (72.7)	69 (82.1)			
	A	36 (27.3)	15 (35.7)	1.725	0.876–3.395	0.115
rs4937333	CC	21 (31.8)	15 (35.7)			
	CT	24 (36.4)	24 (57.1)	0.714	0.299–1.707	0.449
	TT	21 (31.8)	3 (7.1)	5.000	1.259–19.860	0.022
	C	66 (50.0)	54 (64.3)			
	T	66 (50.0)	30 (35.7)	1.800	1.026–3.157	0.040
rs1128355	GG	66 (100)	42 (100)	–	–	–
rs11554584	GG	66 (100)	42 (100)	–	–	–
rs12288765	TT	66 (100)	42 (100)	–	–	–
rs35034701	AA	66 (100)	42 (100)	–	–	–
rs57498864	AA	66 (100)	42 (100)	–	–	–
rs58920409	TT	66 (100)	42 (100)	–	–	–

### mRNA Expression of *ETS1* in PBMCs, CD4+ T Cells, and CD19+ B Cells

**Figure 2:**
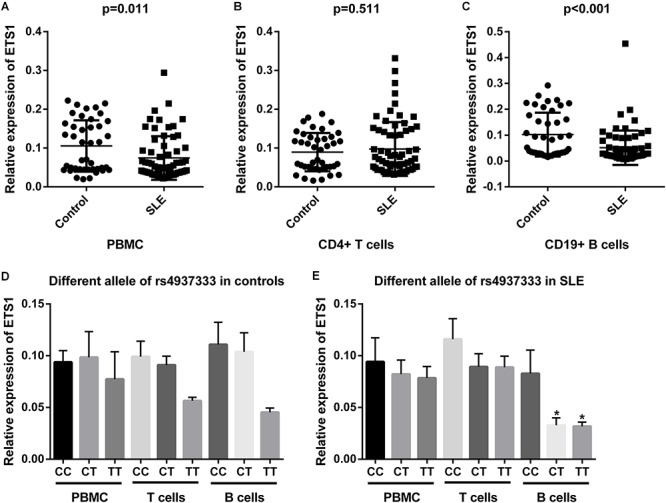
mRNA expression of *ETS1* in **(A)** PBMCs, **(B)** CD4+ T cells, and **(C)** B cells from SLE patients and controls. rs4937333 allele-specific expression of *ETS1* in PBMCs, B cells, and CD4+ T cells in **(D)** controls and **(E)** SLE patients. ^∗^*P* < 0.05 vs. the CC genotype in the same cells.

We examined the mRNA levels of *ETS1* in PBMCs, CD19+ B cells, and CD4+ T cells from 66 SLE patients and 42 healthy controls. Relative *ETS1* mRNA expression in PBMCs was lower in SLE patients than in controls (*P* = 0.011, Figure 2A). We also observed that *ETS1* mRNA levels in CD19+ B cells from SLE patients were lower than those in CD19+ B cells from controls, but no difference in *ETS1* expression in CD4+ T cells was found between the groups (Figure 2B,C).

Next, we determined expression of *ETS1* in SLE patients and controls with different rs4937333 alleles. In PBMCs from controls or SLE patients, no significant differences in *ETS1* mRNA levels were found in any comparisons (Figure 2D). The association of *ETS1* mRNA levels with SNPs was also analyzed in B cells and CD4+ T cells. In B cells from SLE patients, *ETS1* mRNA levels were lower in patients with the TT (*n* = 21) or CT (*n* = 24) genotype than in CC (*n* = 21) homozygotes (Figure 2E). A similar trend was also observed in B cells from controls (Figure 2D). However, no significant associations were found in CD4+ T cells from controls or SLE patients. Thus, carriers of the risk allele T at rs4937333 may exhibit significantly lower *ETS1* mRNA expression in B cells than do individuals with the C allele.

### B Cells With the T Allele in the *ETS1* 3′-UTR Exhibit Enhanced Differentiation Into Plasma Cells

**Figure 3:**
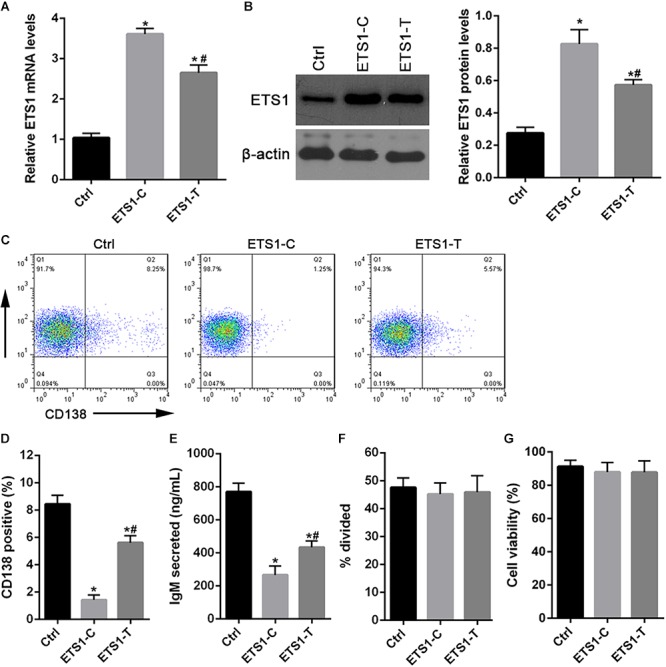
Forced expression of *ETS1* in primary B cells inhibits plasmacytic differentiation. **(A,B)** Lentiviral constructs used for transduction experiments and Western blot analysis of *ETS1* levels in lentivirally infected B cells. **(C)** Flow cytometry analysis of lentivirally infected primary B cells. B cells were stimulated with CpG ODN, infected with lentiviral constructs, and returned to culture in the presence of CpG ODN. **(D)** Two days later, the cells were stained with anti-CD138 antibody and analyzed by flow cytometry to quantify plasma cell differentiation. **(E)** ELISA was performed to assess IgM secretion after GFP+ cells were lentivirally infected and cultured in the presence of CpG ODN. **(F)** Proliferation of CpG ODN-stimulated, GFP+, sorted, virally infected B cells. **(G)** The numbers of live B cells. The data shown are the averages and SE from three independent experiments. ^∗^*P* < 0.05 vs. ctrl; ^#^*P* < 0.05 vs. *ETS1*-C.

To investigate the effect of the SNP rs4937333 on expression of *ETS1*, we drove high-level constitutive expression of *ETS1* using a lentiviral plasmid construct. As expected, cells harboring *ETS1*-C or *ETS1*-T exhibited substantially higher expression of *ETS1* than did cells harboring the control virus (Figure 3A,B).

Then, we tested the effect of the SNP rs4937333 on B cell differentiation. After infection with *ETS1*-T lentivirus or *ETS1*-C lentivirus, B cells were cultured with CpG ODN for 48 h. Then, plasmacytic differentiation was assessed by staining for CD138 (Figure 3C). Cells infected with *ETS1*-C gave rise to four- to fivefold fewer CD138+GFP+ plasma cells than did those infected with *ETS1*-T (Figure 3D). Additionally, forced expression of high levels of *ETS1* suppressed IgM secretion (Figure 3E).

Next, we tested whether B cell proliferation or survival was different between *ETS1*-T and *ETS1*-C B cells. The results shown in Figure 3F,G demonstrate that both proliferation and survival were identical between the genotypes.

### The Allele of the SNP rs4937333 in the *ETS1* 3′-UTR Impacts miR-5003-Mediated Transcriptional Regulation of the *ETS1* Gene

**Figure 4:**
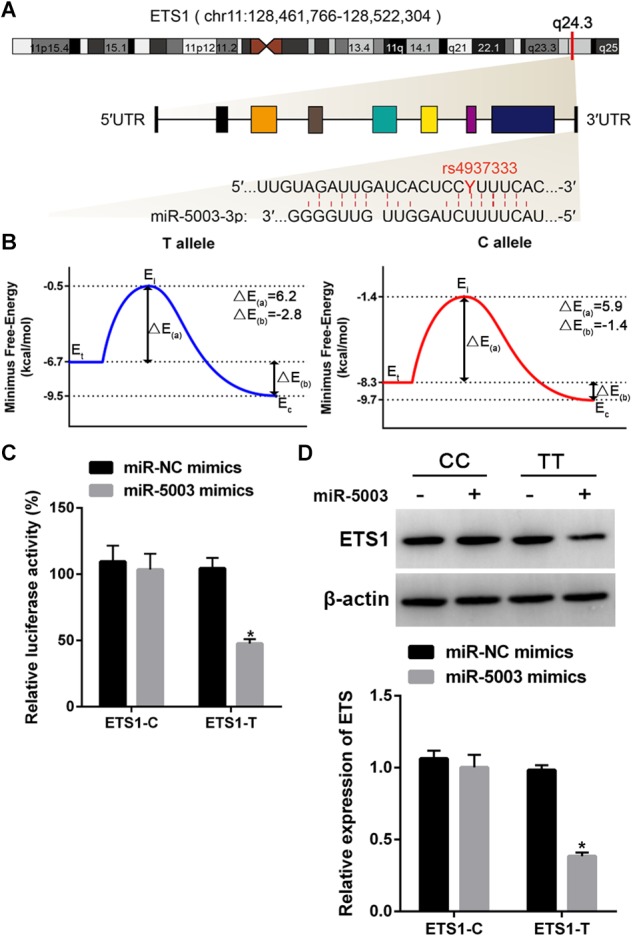
Functional validation of the miR-5003-binding site in the *ETS1* 3′-UTR and the influence of SNP rs4937333. **(A)** *ETS1* gene structure and T/C polymorphism in the *ETS1* 3′-UTR at the miR-5003-binding site. chr, chromosome. **(B)** Binding energy diagram demonstrating the energy transformation of the process of miR-5003 binding to the *ETS1* 3′-UTR. *E*_i_, *E*_intermediate_; *E*_t_, *E*_target_; *E*_c_, *E*_complex_; Δ*E*_(a)_ is the energy difference between the transition state and the original target, and the binding energy, Δ*E*_(b)_, is the energy difference between the complex and the target. **(C)** The effect of the SNP rs4937333 on miR-5003-mediated transcriptional repression in 293T cells. Forty-eight hours after transfection with the reporter gene and miR-5003 mimic, cells with the T allele construct had significantly less relative luciferase activity than in cells with the reporter bearing the C allele. **(D)** The suppressive effect of miR-5003 on *ETS1* gene expression was abolished by rs4937333 C in the *ETS1* 3′-UTR. *ETS1*-T or *ETS1*-C constructs were cotransfected into 293T cells with miR-5003 mimic or miR-NC. *ETS1* expression in 293T cells was analyzed by Western blotting at 48 h after transfection. ^∗^*P* < 0.05 vs. miR-NC; *n* = 3.

Three prediction tools (MirSNP, the PolymiRTS Database 3.0, and MicroSNiPer) showed that the SNP rs4937333 in the *ETS1* 3′-UTR is located in the seed region of the miR-5003-binding site (Figure 4A).

To test whether the SNP rs4937333 could mediate differential regulation of *ETS1* by miR-5003 due to differential binding affinity for the two *ETS1* 3′-UTR genotypes, we used a thermodynamic model to calculate the different energy parameters and then constructed corresponding binding energy diagrams for both 3′-UTR-T (the T allele) and 3′-UTR-C (the C allele) (Figure 4B). The higher energy of the dissociated target (*E*_target_) and smaller activation energy (Δ*E*_(a)_) of the T allele indicated that the T allele was more accessible to miR-5003 than was the C allele variant. The binding energy of the T allele (Δ*E*_(b)_ = -2.8 kcal/mol) was much lower than that of the C allele (Δ*E*_(b′)_ = -1.4 kcal/mol), suggesting that miR-5003 had a higher binding affinity for the T allele.

To further validate the results of computational modeling, a luciferase reporter assay was performed. We observed that the miR-5003 mimic significantly decreased luciferase activity in 293T cells transfected with *ETS1*-T but not *ETS1*-C (Figure 4C).

Additionally, we determined whether rs4937333 affects the expression of *ETS1* in the 293T cell line. The results showed that the miR-5003 mimic markedly reduced *ETS1* expression in *ETS1*-T-transfected cells but did not affect *ETS1* expression in *ETS1*-C-transfected cells (Figure 4D).

These findings indicated that rs4937333 (C→T) could promote miR-5003 binding to *ETS1*, which may contribute to transcriptional repression of *ETS1*.

### Elevated miR-5003 Levels in B Cells

**Figure 5:**
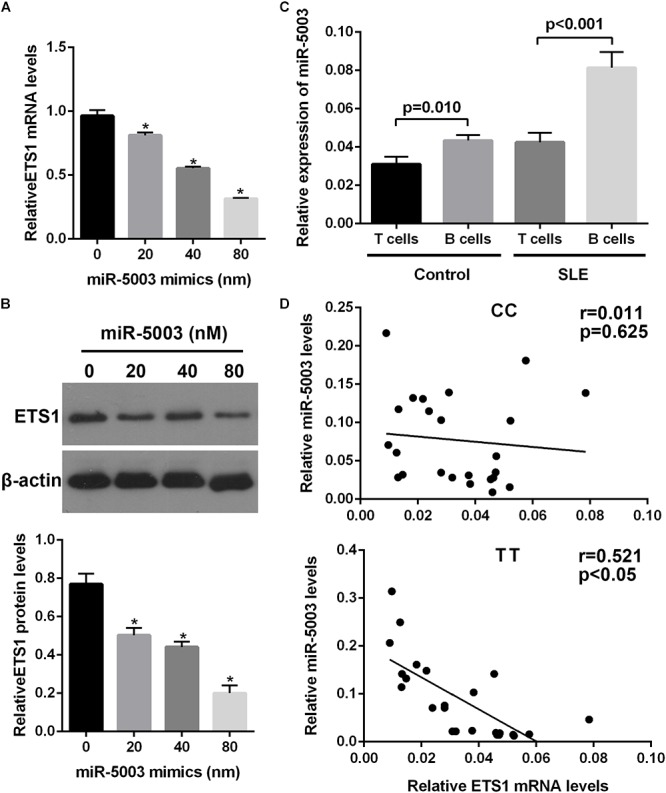
The effect of the variant allele on *ETS1* and miR-5003-3p levels. **(A)** Real-time quantitative polymerase chain reaction (qPCR) analysis of relative *ETS1* mRNA expression in primary B cells transfected with the miR-5003 mimic at the indicated concentrations. **(B)** Western blot analysis of endogenous *ETS1* in primary B cells transfected with miR-5003-3p mimic at the indicated concentrations. **(C)** miR-5003 levels in B cells or T cells of SLE patients and controls. **(D)** Correlation analysis of miR-5003 levels and *ETS1* mRNA levels in B cells from TT or CC carriers. ^∗^*P* < 0.05 vs. miR-NC; *n* = 3.

We examined the potential miR-5003-mediated transcriptional regulation of the *ETS1* gene in B cells with different alleles (the TT and CC genotypes), and the results showed that transfection with the miR-5003 mimic markedly suppressed endogenous *ETS1* expression in B cells with the TT genotype in a concentration-dependent manner but did not affect expression of *ETS1* in B cells with the CC genotype (Figure 5A,B).

We also measured the levels of miR-5003 in T cells and B cells from control subjects or SLE patients using RT-qPCR and found that miR-5003 levels were higher in B cells from SLE patients than in B cells from control subjects. Moreover, miR-5003 levels were higher in B cells than in T cells in SLE patients but not in controls (Figure 5C). Furthermore, a significant negative correlation was observed between miR-5003 levels and *ETS1* mRNA levels in the tested TT carriers (*R*^2^ = 0.255, *P* = 0.001; Figure 5D).

These findings suggested that the reduced levels of *ETS1* associated with the variant allele may be associated with elevated miR-5003 levels.

## Discussion

In the present study, we investigated the associations of SNPs in the 3′-UTR of *ETS1* between SLE subjects and healthy controls. We also investigated the effect of SNPs on the expression level of *ETS1* in T helper cells (CD4+ T cells) and B (CD19+) cells for the first time. We found that rs4937333 of *ETS1* was associated with SLE. In addition, expression of *ETS1* in individuals carrying the rs4937333 T allele was decreased, suggesting a functional explanation for the observed association.

Genetic variants in *ETS1*, including rs1128334, rs4937333, and rs6590330, are associated with SLE and other autoimmune diseases in the Asian population (Han et al., 2009; Yang et al., 2010), but these associations are less well replicated in the Asian population than in European populations (Wang et al., 2013; Morris et al., 2016). In previous studies, common variants of *ETS1* have been found to be significantly associated with SLE in Chinese populations (Guo et al., 2013). In our study, rs4937333 of *ETS1*, but not rs1128334, was significantly associated with the risk of SLE in the Han population in the Kunming area. This discrepancy with respect to rs1128334 may be due to differences in sample sizes and ethnicity.

Several studies have found that *ETS1* mRNA levels are lower in PBMCs from SLE patients than in PBMCs from healthy controls (Garrett-Sinha et al., 2016). Moreover, animal experiments have confirmed that knocking down or knocking out *ETS1* results in lymphocyte abnormalities and loss of self-tolerance in the B cell compartment. (Russell et al., 2015). In our study, we indeed observed lower *ETS1* expression in PBMCs from SLE patients than in PBMCs from controls. Additionally, *ETS1* expression in B cells was significantly lower in SLE patients than in controls, but *ETS1* expression in CD4+ T cells was not different between SLE patients and controls. To eliminate interference from treatment regimens on *ETS1* expression, patients with SLE in the inactive period were selected, and the therapeutic regimens were consistent, with only a low-maintenance dosage of prednisone used within last 3 months. In addition, no evidence shows an effect of prednisone on *ETS1* expression. Therefore, clinical treatment is not expected to affect the expression of the *ETS1* gene in this study.

We further investigated whether the SNP site affected the expression of *ETS1*. However, no difference in *ETS1* expression in PBMCs was observed between healthy individuals with the C allele and those with the T allele. A similar result was observed in SLE patients. We further examined the effects of the SNP on *ETS1* mRNA levels in B cells and CD4+ T cells. Interestingly, *ETS1* levels showed a decreasing trend in samples with the T allele, but a significant difference between the different genotypes was found only in B cells from SLE patients. Few studies have reported the relevance of SNPs to the expression patterns of *ETS1*. In the studies of Yang et al. (2010) and Shan et al. (2014), the A allele of rs1128334 was associated with decreased expression of *ETS1*.Wei et al. (2014) found that *ETS1* expression in rs10893872 CC carriers was significantly higher than that in CT and TT individuals. Since the SNPs rs10893872 and rs4937333 have absolute LD with each other (Yang et al., 2010), expression of *ETS1* with the T allele was reduced compared to that with the C allele. In our study, the results for rs4937333 are in accordance with those reported in the above studies. Because no association was found between rs1128334 and SLE, we did not investigate this allelic expression of *ETS1*.

In general, *ETS1* functions to regulate B cell differentiation to plasma cell. Thus, the effect of the SNP on B cell differentiation was investigated. We observed that the differentiation of *ETS1*-C B cells into IgM-secreting cells was decreased in the presence of CpG ODN. *ETS1* did not have a major impact on proliferation or survival, but alterations in CD138+ cell numbers and IgM secretion indicated that the SNP could affect B cell differentiation into plasma cells. John et al. (2008) found that purified splenic B cells isolated from *ETS1*-deficient mice exhibit greater terminal differentiation into IgM-secreting cells than those isolated from non-deficient mice, and that forced expression of *ETS1* could block efficient differentiation into CD138 plasma cells and secretion of IgM.

Accumulating evidence has demonstrated that miRNAs can regulate target gene expression and affect mRNA translation into proteins through sequence-specific binding to the 3′-UTRs of target mRNAs (Ambros, 2004). The binding of miRNAs to target genes can be affected by SNPs residing in the miRNAs or in the target gene 3′-UTRs (Moszynska et al., 2017). Indeed, the effect of SNPs on binding affinity between miRNAs and target genes has been proposed to contribute to susceptibility to autoimmune diseases (Baulina et al., 2016). rs4937333 is located in the 3′-UTR of *ETS1* and may affect expression of *ETS1* via miRNA-binding activity. Therefore, we screened the miRNAs that bind to *ETS1* at rs4937333. Three bioinformatics software programs showed that binding between miR-5003-3p and *ETS1* was increased by the C to T mutation in rs4937333. Computational analysis revealed that the presence of a T allele in the *ETS1* gene increased the binding affinity of miR-5003-3p to the target RNA. Thus, rs4937333 variants may enhance miR-5003 binding and suppress *ETS1* gene expression as determined by a reporter assay.

Additionally, we demonstrated that miR-5003 effectively inhibited the mRNA and protein expression levels of *ETS1*-T and that this effect was dose-dependent in B cells. Since an effect of rs4937333 on *ETS1* expression was observed in B cells from SLE patients but not in PBMC or CD4+ T cells, we hypothesized that miR-5003 was differentially expressed between B cells and T cells. As expected, the expression level of miR-5003 in B cells was significantly higher than that in T cells in both healthy subjects and SLE patients. Furthermore, a negative correlation between *ETS1* mRNA levels and miR-5003 was observed in B cells. Therefore, high levels of miR-5003 were able to inhibit *ETS1* expression more significantly with the enhanced binding to *ETS1* mediated by the T allele. However, the effect of high miR-5003 expression in B cells still requires further study.

Unfortunately, there are some limitations taken into account in this research. The sample size for ETS1 expression and gene polymorphism detection is small, which may reduce the power of the study. And, we did not analyze the correlation between ETS1 and clinical data for the reason that SLE patients in the inactive phase were chosen, and the therapeutic regimen was consistent. Moreover, the association of autoimmune diseases and SNPs in ETS1 gene is inconsistent in the epidemiologic literature, besides we only measured the rs4937333 SNP that might be associated with SLE. More SNPs in the 3′-UTR of ETS1 genes and a larger sample should be conducted to confirm our observations in future research. In addition, positively selected T cells were only used for qPCR, rather than any functional analysis, assuming the mRNA level is insensitive to positive selection. Whether positive selection affects the ETS1 expression could be further examined in the following studies.

In summary, our data demonstrated that rs4937333 plays an important role in SLE susceptibility, modulating the epigenetic regulation of a critical SLE-related gene, *ETS1*, especially in B cells, rs4937333 impairs the binding of miR-5003-3p to *ETS1* mRNA, which leads to enhanced differentiation of B cells into plasma cells. However, the impact of the *ETS1* SNP on the development and function of SLE remains incompletely understood, and further exploration of the regulatory mechanism is warranted.

## Ethics Statement

This study was carried out in accordance with the recommendations of “the ethical standards described in the 1964 Declaration of Helsinki, the Institutional Ethics Committee of Kunming Medical University”. All subjects gave written informed consent in accordance with the Declaration of Helsinki. The protocol was approved by the Ethics Committee of the Second Affiliated Hospital of Kunming Medical University. And the authorization number is Shen-PJ-2018-40.

## Author Contributions

XL: study conception, design, and critical revision. RZ, BP, and YL: acquisition of data. BP: analysis. RZ: drafting of the manuscript. All authors were involved in the interpretation of the data.

## Conflict of Interest Statement

The authors declare that the research was conducted in the absence of any commercial or financial relationships that could be construed as a potential conflict of interest.
