# Supplementary material for: SNP rs4937333 in the miRNA-5003-Binding Site of the ETS1 3′-UTR Decreases ETS1 Expression
Source: Front Genet. 2019 Jun 19;10:581. doi: 10.3389/fgene.2019.00581 (PMC6593064; doi:10.3389/fgene.2019.00581)
Supplement: Supplementary file 1 [file Table_1.DOC]

**Table S1. The primer sequences used for genotyping of the SNPs and qPCR of miRNA or ETS1**

| Name | Sequence **(5’-3’)** | Annealing  temperature |
| --- | --- | --- |
| ETS1 | TACACAGGCAGTGGACCAATC | 60℃ |
|  | CCCCGCTGTCTTGTGGATG |  |
| GAPDH | CCAGGTGGTCTCCTCTGACTT | 60℃ |
|  | GTTGCTGTAGCCAAATTCGTTGT |  |
| miR-5003 | TCACAACAACCTTGCAGGGT | 60℃ |
|  | Universal primer |  |
| rs4937333**,** rs12288765**,** rs35034701 | TTTTGAGAGCTTCATTAAGGC | 57℃ |
|  | TCAATAAAATGTGACTTGGCTG |  |
| rs57498864 | AAATTTAATAGGAACGGTGGAT | 57℃ |
|  | CAAAGGCAGAATTATCCCAG |  |
| **rs1128334, rs1128355, rs11554584,** rs58920409 | CGCTTACTCTGTTGGGGTCTATA | 58℃ |
|  | TAAACTGCATCCCTCTGTGCT |  |
